# Nanohydroxyapatite Electrodeposition onto Electrospun Nanofibers: Technique Overview and Tissue Engineering Applications

**DOI:** 10.3390/bioengineering8110151

**Published:** 2021-10-22

**Authors:** Thiago Domingues Stocco, Pedro José Gomes Rodrigues, Mauricio Augusto de Almeida Filho, Anderson Oliveira Lobo

**Affiliations:** 1 Faculty of Medical Sciences, State University of Campinas, Campinas 13083-877, Brazil; tdstocco@live.com; 2 Faculty of Physiotherapy, University of Santo Amaro, São Paulo 04829-300, Brazil; 3 LIMAV—Interdisciplinary Laboratory for Advanced Materials, BioMatLab, UFPI—Federal University of Piauí, Teresina 64049-550, Brazil; pedrojgr@ufpi.edu.br (P.J.G.R.); maufilho@hotmail.com (M.A.d.A.F.)

**Keywords:** nanohydroxyapatite, electrodeposition, polymeric nanofibers, scaffold, tissue engineering

## Abstract

Nanocomposite scaffolds based on the combination of polymeric nanofibers with nanohydroxyapatite are a promising approach within tissue engineering. With this strategy, it is possible to synthesize nanobiomaterials that combine the well-known benefits and advantages of polymer-based nanofibers with the osteointegrative, osteoinductive, and osteoconductive properties of nanohydroxyapatite, generating scaffolds with great potential for applications in regenerative medicine, especially as support for bone growth and regeneration. However, as efficiently incorporating nanohydroxyapatite into polymeric nanofibers is still a challenge, new methodologies have emerged for this purpose, such as electrodeposition, a fast, low-cost, adjustable, and reproducible technique capable of depositing coatings of nanohydroxyapatite on the outside of fibers, to improve scaffold bioactivity and cell–biomaterial interactions. In this short review paper, we provide an overview of the electrodeposition method, as well as a detailed discussion about the process of electrodepositing nanohydroxyapatite on the surface of polymer electrospun nanofibers. In addition, we present the main findings of the recent applications of polymeric micro/nanofibrous scaffolds coated with electrodeposited nanohydroxyapatite in tissue engineering. In conclusion, comments are provided about the future direction of nanohydroxyapatite electrodeposition onto polymeric nanofibers.

## 1. Introduction

Recent progress in regenerative medicine, especially in tissue engineering, have provided new treatment options for a number of conditions and diseases. Many of these advances are directly attributable to the development of more suitable and effective scaffolds for tissue regeneration [1,2]. Fundamentally, tissue engineering is based on the combination of living cells, growth-stimulating signals, and biomaterial scaffolds to produce engineered structures able to promote regeneration or replace diseased or damaged tissues [3].

Although different types of human tissues have specific demands within tissue engineering, in general, the synthesis of an ideal scaffold should take into account the physical, chemical, and biological properties of the native extracellular matrix, to mimic the microstructure and function of the original tissue. The scaffold should promote an ideal environment to stimulate, guide, and support the growth of the new tissue [4,5].

The convergence of nanotechnology with biomedical sciences has revolutionized the tissue engineering field. The potential to create structures with nanoscale components has allowed the synthesis of innovative biomimetic scaffolds [6]. The main applications of nanostructured materials include the development of scaffolds from polymeric nanofibers. Nanofiber-based scaffolds are able to recapitulate the major structural components of the native extracellular matrix, simulating the original environment, persuading cells to behave in the same way as native tissue [7].

The versatility allowed during the synthesis of polymeric nanofibers, which can be produced using a simple, inexpensive, and adjustable tool called electrospinning, permits the construction of a multitude of scaffold configurations for a wide range of applications [7]. In addition to the usual choice between the broad range of synthetic and natural polymers, researchers have incorporated other biomaterials to produce nanofibrous polymer scaffolds with improved mechanical and biological properties, thereby generating nanocomposites. Nanocomposite-based scaffolds are generically described as heterogeneous structures composed of two or more biomaterials, with the prefix “nano” being attributed to the presence of at least one nanoscale component [7,8].

In recent years, the incorporation of nanofillers in nanofibrous scaffolds has gained considerable attention. Among the main nanobiomaterials used as nanofillers in tissue engineering is nanohydroxyapatite (nHAp). This type of inorganic nanomaterial presents unique characteristics, such as excellent bioactivity, biocompatibility, and osteoconductive capacity. In addition, nHAp presents important bone integration ability and similarity to the major mineral component of natural bone tissue and teeth, which make it especially important for the regeneration of these tissues [9,10].

Although several attempts to incorporate nHAp particles into polymeric nanofibers have been successfully carried out, through this manufacturing strategy, nHAp becomes available to the organism slowly, since it depends on the previous degradation of the polymer [11,12,13,14,15]. Given the above, externally coating polymeric nanofibers with nHAp has shown to be a more promising approach to improving their bioactivity.

An exciting way to produce nHAp-coated nanofibers is with electrodeposition. The electrodeposition technique has attracted considerable interest, as it allows quick and efficient preparation of thin, adherent, and homogeneous coatings, usually using low-cost equipment [16,17]. Within the scope of the current mini-review, nanofibrous polymer scaffolds coated with electrodeposited nHAp are discussed. In particular, the role of nHAp coatings is described and the method of electrodepositing nHAp onto polymer electrospun nanofibers is detailed. Finally, we present the main findings regarding the application of nanofibrous scaffolds coated with electrodeposited nHAp in tissue engineering.

## 2. Rationale for Using nHAp as a Coating in Tissue-Regenerative Scaffolds

It is widely discussed and known that the interactions between cells and scaffolds are primarily affected by the physical–chemical properties of the surface of the biomaterial [18]. Therefore, modifications on the scaffold surface have been performed to obtain more adequate structures that promote tissue regeneration. A classic approach is to coat the scaffolds with bioactive materials such as hydroxyapatite (HAp) [19].

**Figure 1:**
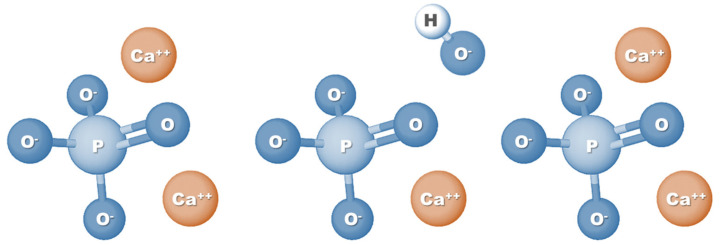
Chemical formula structure of hydroxyapatite.

HAp is a calcium phosphate-based material and corresponds to the main mineral component of teeth and bones. Specifically, HAp represents 60–70% of human bone tissue, which is one of the main reasons why this biomaterial is so widely used in tissue engineering applications, especially for bone regeneration [9]. Also known as hydroxylapatite, HAp can be represented by the formula Ca_5_(PO_4_)_3_(OH) (Figure 1); however, since the crystal unit cell includes two entities, it has typically been expressed by the chemical formula Ca_10_(PO_4_)_6_(OH)_2_ [20]. The molar ratio between calcium and phosphorus (Ca/P ratio) stoichiometric HAp is 1.67; nevertheless, HA found naturally in human bone tissue is generally not stoichiometric and has a Ca/P ratio ranging between 1.5 and 1.67. This difference in molar ratio, which can also be seen when comparing synthetic HAp with natural HAp, can directly affect the physical and biological properties of HA [21,22].

Because of its chemical similarity to native bone tissue, HAp has properties that make it capable of promoting mineralization and accelerating the bone healing process by osteoconduction and osteoinduction, without causing inflammation or adverse reactions in the human body [23,24,25].

The HAp surface has been shown to be especially interesting for supporting bone cell adhesion, growth, and differentiation. As HAp consists of only phosphate and calcium ions, it does not induce local or systemic toxicity or stimulate a foreign body reaction, making it an optimal bioactive material for bone tissue engineering [23]. Osteoinduction, the process by which host mesenchymal stem cells are stimulated to differentiate into bone-forming osteoblastic cells, and which plays a crucial role in bone regeneration, has been observed extensively in studies using HAp [23,26,27,28]. In addition, in contact with physiological fluids, HAp forms strong bonds with both soft and hard body tissues, thus stimulating osteointegration [24,25]. Implants coated externally with HAp exhibit better bone fixation, greater osteoblastic activity, more bioactivity, and longer lifetime [29,30]. Finally, in vivo studies have demonstrated the fastest and most adequate bone regeneration around HAp-coated implants [31,32,33].

Advances in the nanotechnology field and in the manufacture of nanostructured biomaterials to mimic complex nanoscale characteristics of native bone have led to the development of nHAp. Nanobiomaterials can further stimulate bone growth compared to conventional materials, mostly because they have a larger surface area and, consequently, higher reactivity [34,35]. Furthermore, it has been described that cell morphology is more affected by nanotopography than by chemical composition [36,37].

In comparison to standard HAp, the nanoscale characteristics of nHAp promote advantageous cellular responses and induce greater amounts of specific protein interactions [25]. nHAp has the ability to promote more cell adhesion and proliferation (e.g., osteoblasts) than identical biomaterials without nanostructure [38]. Additionally, when incorporated into polymer matrix scaffolds, nHAp exhibited more osteoconductive properties than HAp microparticles (equal concentration) [25].

The nHAp coating can provide scaffolds with a large surface area and high energy, strong protein adsorption capacity, and adequate wettability and roughness on a nanoscale, increasing the performance of bone cells and favoring osteointegration [39,40,41,42]. Given the above, coating tissue-regenerative scaffolds with nHAp provides unique benefits for bone tissue engineering.

## 3. nHAp Electrodeposition Method

Several approaches have been developed to incorporate nHAp on the surface of tissue-engineered scaffolds, including hydrothermal process [43], plasma spray technique [44], ultrasonic irradiation [45], and electrospray [46], as well as the main focus of this study, electrodeposition, which is a simple, versatile, low-cost, widely used, and developed surface modification method that offers promising possibilities for the fabrication of nHAp coatings [17,47,48].

### 3.1. Method Overview

Electrodeposition refers to an efficient technique to synthesize solid coating (film) on the surface of a conductive material from electrochemical reactions. This technique can be employed to precisely control the chemical composition, thickness, and structure of electrodeposited material, generating more uniform, crystalline, homogeneous, adherent, and thin coatings via a fast, adjustable process at a relatively low temperature [17,49,50,51].

**Figure 2:**
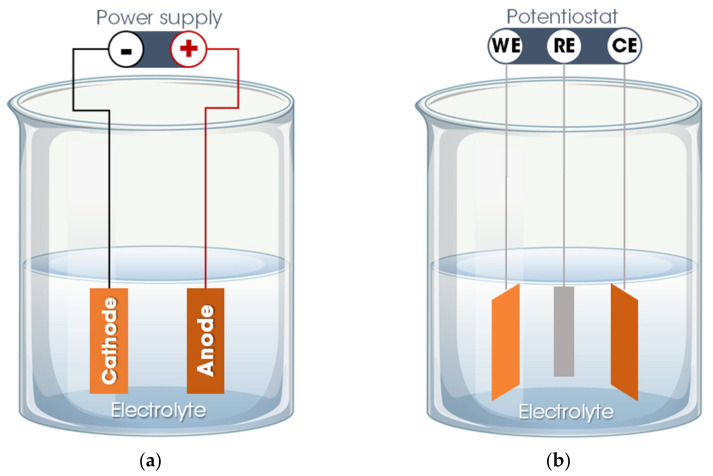
Electrodeposition system: (**a**) Two-electrode system for galvanostatic electrodeposition and (**b**) three-electrode system for potentiostatic electrodeposition. Legend: WE = working electrode; CE = counter electrode; RE = reference electrode.

Basically, the electrodeposition process is obtained by passing an electric current between two or more separate electrodes immersed in an electrolyte solution. Thus, a basic electrodeposition system contains a cathode (negative electrode), an anode (positive electrode), an electrical power supply, and an aqueous electrolytic solution that provides the elements of the material to be deposited or works only as a source of ions to close the electrical circuit and promote the transfer of material from one electrode to another [50,52]. The electrode to which the material is directed, functioning as a substrate for the formation of the coating, is called the working electrode (WE). The other electrode, defined as a counter electrode (CE), can be an inert material (e.g., platinum) or an active material that provides elements for the synthesis of the film. Occasionally, a third electrode is incorporated into the electrodeposition system—the reference electrode (RE), which measures the potential of the working electrode. Thus, the electrodeposition process is categorized into two setups: A traditional two-electrode system, used for the galvanostatic electrodeposition mode in which the current is fixed, and the potential varies according to the requirements of the electrodeposition process (Figure 2a), and in a three-electrode system in which a constant potential is applied, allowing a variation of the current, defined as the potentiostatic electrodeposition mode (Figure 2b) [50,51].

Specifically, for the formation of the HAp coating, the aqueous electrolyte solution of the electrodeposition process is based on phosphate ions and calcium ions (or hydrogen phosphate/dihydrogen phosphate ions) and the basic chemical reactions involved in the process are described as follows:

(1) $$2H_{2}O+2e^{-}\to {\;H}_{2}↑\;+\;2{\mathrm{OH}}^{-}$$

(2) $$H_{2}{\mathrm{PO}}_{4}^{-}+{\mathrm{OH}}^{-}\;\to {\mathrm{HPO}}_{4}^{2-}+{\;H}_{2}O$$

(3) $${\mathrm{HPO}}_{4}^{2-}+{\mathrm{OH}}^{-}\;\to {\;\mathrm{PO}}_{4}^{3-}\;+{\;H}_{2}O$$

(4) $$10{\mathrm{Ca}}^{2+}+6{\mathrm{PO}}_{4}^{3-}+\;2{\mathrm{OH}}^{-}\;\to {\mathrm{Ca}}_{10}{\left({\mathrm{PO}}_{4}\right)}_{6}{\left(\mathrm{OH}\right)}_{2}$$

Equation (1) shows the production of hydrogen (which can be observed by the presence of bubbles close to the cathode) and the generation of hydroxyl radicals, which causes the pH value near the cathode to increase. In addition, as presented in Equations (2) and (3), both the concentrations of phosphate ions and hydrogen phosphate ions increase as a result of a higher concentration of hydroxide ions. Then, concurrently, the calcium ions are directed to the cathode, where they interact with the phosphate group and hydroxyl radicals, generating HAp (Equation (4)) [51,53].

The properties of the formed HAp coating, such as crystallinity, morphology, thickness, porosity, and nanostructure of the coating are directly influenced by the electrodeposition parameters and variables, including electrolyte composition and concentration, current type and density, temperature, pH, and deposition time. For instance, increasing the deposition time gradually increases the Ca/P ratio of the HAp coating and the degree of crystallinity, as well as improving roughness [53]. The variation in temperature also changes the properties of the coating. A high deposition temperature (75 °C) is beneficial for generating denser, more compact HAp coatings, with a high degree of crystallization, greater hydrophilicity, and thinner crystal morphology [54]. Another example is in relation to the pH value of the electrolyte. Adjusting the pH of the electrolyte from 5 to 6 results in greater resistance to corrosion and greater capacity for biomineralization of HAp coatings [55]. The electric current type applied during electrodeposition also changes the final result. HAp coatings can be electrodeposited using different forms of current, including continuous direct current, pulsed current, or pulsed reverse current, with significant morphological and mechanical differences in the material formed [51]. Regarding the composition of the electrolyte, in addition to the wide variety of precursors of phosphate (NH_4_H_2_PO_4_ [56], (NH_4_)_2_HPO_4_ [57], and KH_2_PO_4_ [58]) and calcium (CaCl_2_ [58], Ca(NO_3_)_2_ [59], and Ca(NO_4_)_2_ [60]) in their different concentrations, some additives are commonly incorporated into the electrolyte during the electrodeposition of HAp, each with a specific purpose—for example, the addition of NaCl to increase electrolytic conductivity [61], the incorporation of NaNO_3_ to improve ionic strength [62], and the inclusion of H_2_O_2_ that contributes to the formation of hydroxyl ions favoring the formation of HAp [58]. A detailed discussion of the influence of each of these variables on the electrodeposition process of HAp can be found in a recently published review about this topic [49].

### 3.2. nHAp Electrodeposition onto Polymeric Nanofibers

Although it may be used alone as a scaffold, nHAp is most commonly applied as a component within a polymeric matrix, forming a nanocomposite. Nanocomposites that integrate nHAp and nanofibers from biocompatible polymers can be optimized to capture the properties and benefits of both components. However, adequately incorporating relevant amounts of nHAp into polymeric nanofibers is still a challenge to be overcome. This is because the main technique for preparing these nanocomposites is based on the electrospinning to blend nHAp with the polymeric solution [12,15,63,64]. The simple mixing of nHAp particles previously synthesized with polymers usually results in a nanocomposite with limited properties and several problems, including the agglomeration and poor dispersion of nHAp powder, which leads to a reduction in the efficiency of the electrospinning process and the construction of mechanically weak scaffolds. In addition, the presence of nHAp on the fiber surface is minimal, thus impairing osteoconductivity and decreasing the bioactivity of the scaffold [25,65,66].

In this context, the electrodeposition of the nHAp coating on the surfaces of polymeric nanofibers emerges as a hopeful strategy to help resolve these issues. Based on the expertise of our research group with nHAp electrodeposition [16,17,47,67], we developed a protocol to incorporate nHAp particles onto the surface of ultrathin polymer fibers [68,69,70].

The first step is the synthesis of polymer-based nanofibers. Although several techniques are available for the production of ultrathin fibers from biocompatible polymers (e.g., rotary-jet spinning, airbrush, and self-assembly), electrospinning has proven to be the most popular method since it is a low-cost, simple, versatile, and powerful tool for this purpose. A basic electrospinning setup consists of a few key components: A syringe loaded with a polymeric solution (with a metallic needle) attached to a syringe pump, a high voltage power supply, and a collector (usually electrically grounded). The system is configured so that a high voltage electric field is established between the needle and the collector. Briefly, during electrospinning, the solution is gradually expelled from the syringe forming a drop of polymeric solution at the end of the needle (maintained by its surface tension). As the intensity of the electric field increases, this drop of solution extends to form a cone, called the Taylor cone. At a certain value of the intensity of the electric field in which the repulsive electrostatic force exceeds the surface tension, a jet of solution is ejected toward the collector (while the solvent evaporates), where it accumulates in the form of ultrafine fibers [7]. It is important to highlight that, in the case of nanofibers synthesized to be coated with nHAp via electrodeposition, in addition to the traditional criteria for choosing which polymer will be used to build the scaffold (e.g., biocompatibility, bioactivity, bioresorbable, and mechanical properties), conductive properties must be especially taken into account.

The second stage involves precisely the electrodeposition of nHAp on the surface of the polymer fiber scaffolds. For this, a three-electrode system is utilized in which the nanofibrous scaffold produced in the previous step is used as WE, inserting it into a sample holder (copper/Teflon electrochemical cell with a geometric area exposed to the solution of ~0.28 cm^2^). As CE, a platinum rod is used, while Ag/AgCl (3 M KCl _(aq.)_) is employed as RE. To maintain the temperature, constant agitation, and pH of the electrolyte, the electrodeposition process must be performed with the support of a thermostatic bath, magnetic stirring, and a real-time pH meter.

**Figure 3:**
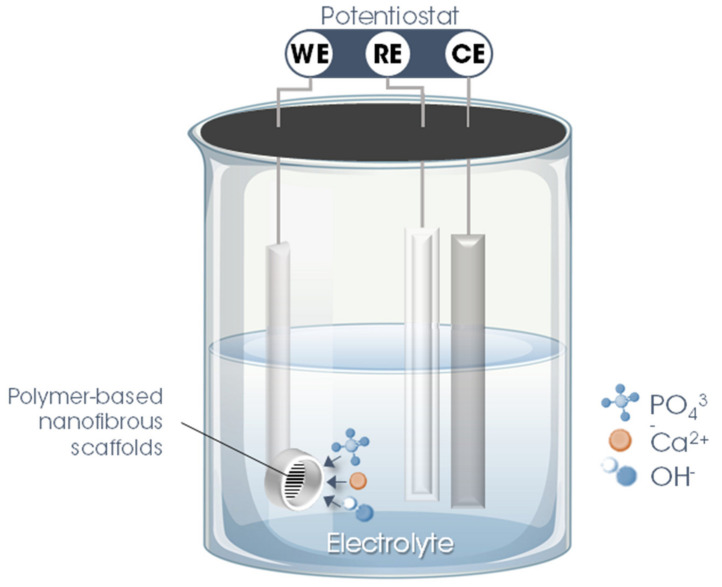
Schematic illustration of the hydroxyapatite electrodeposition process on the surface of electrospun polymer nanofibers. Legend: WE = working electrode; CE = counter electrode; RE = reference electrode.

The parameters and variables of the nHAp electrodeposition process onto polymer-based nanofibrous scaffolds are described in detail in Table 1 and illustrated in Figure 3.

## 4. Applications of Nanofibrous Scaffolds Coated with Electrodeposited nHAp

**Figure 4:**
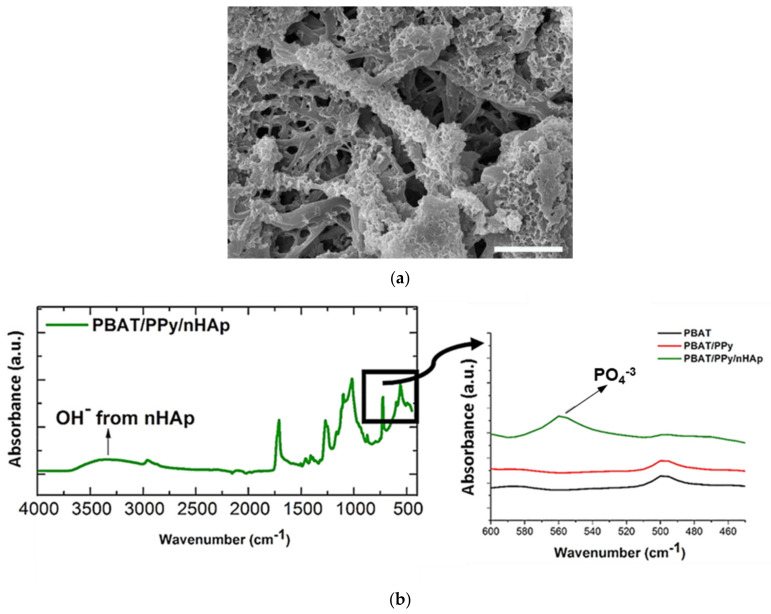
(**a**) FE-SEM micrographs of nHAp crystals electrodeposited onto the PBAT/PPy scaffold surface (scale bar indicates 2.5 μm); (**b**) ATR-FTIR spectra of PBAT/PPy/nHAp indicating the absorption of OH^–^ (vibrational band in the 3500 cm^−1^ range) and PO_4_^−3^ (absorption peak at 566 cm^−1^). Reproduced with permission from ref. [68]. Copyright 2016 The Royal Society of Chemistry.

As a consequence of its high similarity to the mineral phase of natural bone and unique properties capable of accelerating the healing process of bone tissue, obviously, the main application of nHAp has been bone repair [9,10,71]. This scenario is the same when considering the application of scaffolds based on polymeric fibers coated with electrodeposited nHAp. In 2016, Castro et al., from our research group, were the first to synthesize and investigate this type of scaffold [68]. The authors electrodeposited nHAp crystals effectively on the surface of electrospun polymer fibers (132 nm, fiber average diameter) based on a blend of polybutylene adipate terephthalate (PBAT) and polypyrrole (PPy), a biocompatible and conductive polymer, which was incorporated primarily to provide electrical conductivity to the material. The nHAp electrodeposition was confirmed by field-emission scanning electronic microscopy (FE-SEM), through which it is possible to view HAp nanocrystals homogeneously deposited onto the PBAT/PPy nanofibers (Figure 4a); and via attenuated total reflectance Fourier transform infrared spectroscopy (ATR-FTIR) represented by the PO_4_^3−^ absorption peak at 566 cm^−1^ and by the vibrational band in the region of 3500 cm^−1^ (OH^−^ absorption peak; Figure 4b). In vitro biological assays, which evaluate the performance of human osteoblasts from the MG-63 cell line by scanning electronic microscopy (SEM), 3-(4,5-dimethylthiazol-2-yl)-2,5-diphenyltetrazoliumbromide (MTT) colorimetric method and alkaline phosphatase (ALP) assay demonstrated that the novel PBAT/PPy/nHAp hybrid scaffold provides an appropriate surface for adhesion without a cytotoxic effect and stimulates osteoblastic differentiation.

**Figure 5:**
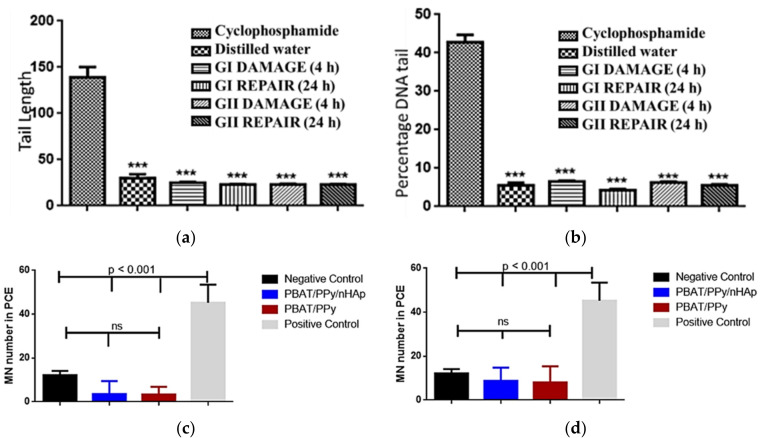
No in vivo genotoxic effects of the PBAT/PPy/nHAp scaffold were detected. (**a**) Tail length and (**b**) percent DNA tail of comet assay of the animals after exposure. Mean of micronuclei found after (**c**) acute (after 48 h) and (**d**) chronic (after 72 h) exposure of the animal to the scaffold. Legend: cyclophosphamide = positive control; distilled water = negative control; GI = PBAT/PPY/nHAp; GII = PBAT/PPy; ***: *p* < 0.001 [69].

The excellent properties of the PBAT/PPy/nHAp nanocomposite were confirmed via in vivo animal tests [69]. The scaffolds implanted in Wistar rats did not present genotoxic effects, analyzed by comet tests (Figure 5a) and micronucleus assays (Figure 5b) after acute and chronic exposure. Peripheral blood samples collected from the animals evidenced that the PBAT/PPy/nHAp scaffolds did not induce damage to the percent DNA tail or for tail length, highlighting the promising potential of this new nanocomposite as a scaffold for bone tissue engineering application.

**Figure 6:**
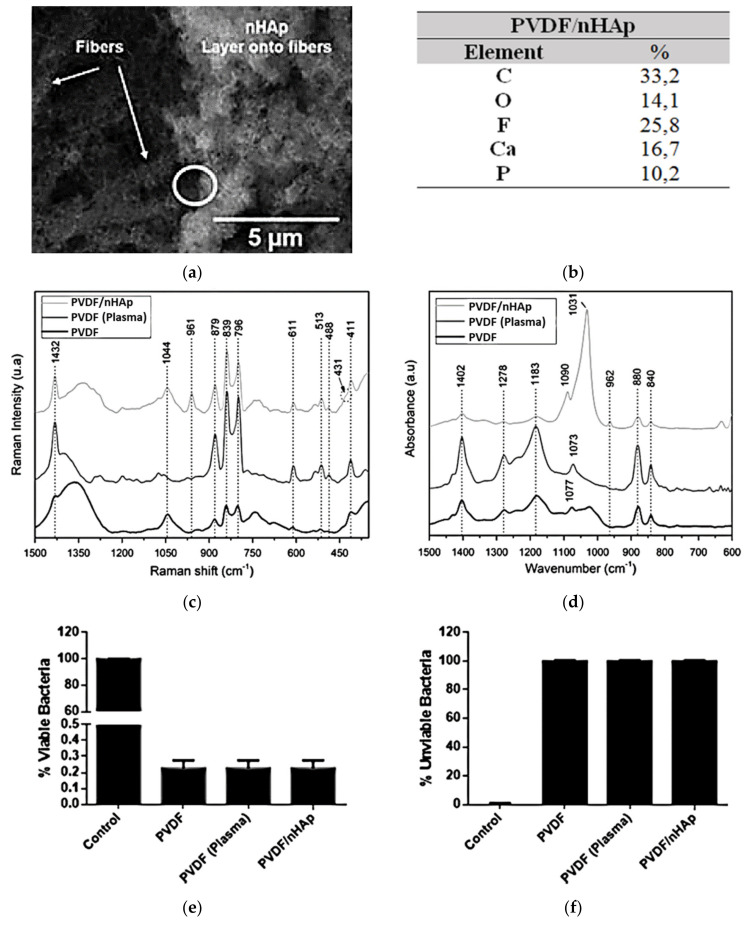
nHAp electrodeposition onto electrospun PVDF nanofibers illustrated in (**a**) SEM micrographs and confirmed by (**b**) the chemical composition obtained from the EDS spectra (Ca/P ratio = 1.64), and by typical bands and peaks attributed to PVDF and nHAp viewed using (**c**) Raman spectroscopy spectra and (**d**) ATR-FTIR spectra. The excellent bactericidal effect of the PVDF/nHAp scaffold is demonstrated in the graphs of proliferation of (**e**) viable bacteria and (**f**) unviable bacteria. Reprinted with permission [70].

Recently, by the same strategy, our research group developed a novel polyvinylidene fluoride (PVDF)/nHAp nanobiomaterial aimed at application in bone regenerative medicine [70]. First, nanofibrous PVDF-based scaffolds were obtained by the electrospinning technique (133.6 nm, fiber average diameter). The choice of PVDF is related to its biocompatibility, ease of fabrication, good performance with the stimulation of bone regeneration, and osteogenic differentiation, and principally due to its excellent piezoelectricity [72,73,74,75,76]. Thus, for the synthesis of PVDF nanofiber meshes with electroactive behavior, nHAp coating was accurately electrodeposited on the surface of the scaffold (202.6 nm, average diameter of the deposited nHAp) (confirmed via SEM, energy-dispersive X-ray spectrometry (EDS) spectrum, Raman spectroscopy, and ATR-FTIR; Figure 6a–d). When the PVDF/nHAp nanocomposite was seeded on MG-63 human osteoblast-like cells and analyzed for cell viability, total protein content, and ALP activity after seven days, the results elucidated the potential of this novel bioactive scaffold for bone tissue engineering applications. In addition, the bacteria *Pseudomonas aeruginosa* were inoculated on the scaffold to assess its bactericidal activity. The bacterial growth was reduced substantially (Gram-positive and -negative bacteria), indicating a high efficiency of the scaffold against this type of bacteria (Figure 6e,f).

## 5. Final Considerations and Future Perspectives

This report shows that nHAp electrodeposition onto polymeric nanofibers is an attractive and feasible strategy for surface modification of scaffolds and synthesis of nanocomposites toward bone regenerative medicine, combining the excellent properties of polymers with the unique characteristics of nHAp. Nevertheless, the findings reveal some weaknesses that need to be highlighted. To date, few polymers (PPy, PBAT, and PVDF) have been researched for nHAp electrodeposition onto polymeric nanofibers. Future studies should also focus on the electrodeposition of an nHAp coating on other conductive biocompatible polymers, including poly(3-hydroxybutyrate-co-3-hydroxyvalerate) (PHBV), polyaniline (PANI), polythiophenes (PT), or non-conductive polymers, but which have electrical conductivity provided by the incorporation of other nanobiomaterials, such as carbon nanotubes and graphene.

The optimal coating thickness should also be further investigated in future works. The literature reports the ideal thickness of HAp coating for osteogenesis (~10 µm) [49]; nevertheless, this parameter has not been explored in studies that conducted nHAp electrodeposition on polymeric nanofibers.

Although polymer-based electrospun fibers and nHAp have attracted considerable attention in the field of dentistry [35,77,78,79], the electrodeposition of nHAp crystals as a coating for polymeric nanofibers remains underexplored for this purpose. This new methodology may present a promising strategy for the construction of scaffolds and implants for future applications in dental tissue engineering and thus justifies further investigations.

Moreover, owing to the novelty of this method, further studies should be performed to confirm the efficacy and biosecurity of these novel nanocomposite scaffolds prior to the transition from basic research to widespread clinical application.

## Figures and Tables

**Table 1:** Electrodeposition parameters adjusted for the formation of nHAp coating on the surface of polymer-based nanofibrous scaffolds.

**Electrodeposition Parameters**	**Values for the Deposition of nHAp in Polymeric Nanofibers**
Electrodeposition mode	Potentiostatic
Calcium precursor/concentration	Ca(NO_3_)_2_·4H_2_O/0.042 mol L^–1^
Phosphate precursor/concentration	NH_4_H_2_PO_4_/0.025 mol L^–1^
Ca/P ratio	1.67
Temperature	~70 °C
pH value	4.7–5.0
Deposition time	30 min
Potential	−3.8 V
